# Profiling the Location and Extent of Musicians’ Pain Using Digital Pain Drawings

**DOI:** 10.1111/papr.12581

**Published:** 2017-05-28

**Authors:** Cinzia Cruder, Deborah Falla, Francesca Mangili, Laura Azzimonti, Liliana S. Araújo, Aaron Williamon, Marco Barbero

**Affiliations:** ^1^ Department of Research and Development Conservatory of Southern Switzerland Lugano Switzerland; ^2^ Rehabilitation Research Laboratory (2rLab) Department of Business Economics, Health and Social Care University of Applied Sciences and Arts of Southern Switzerland Manno Switzerland; ^3^ Centre of Precision Rehabilitation for Spinal Pain (CPR Spine) School of Sport, Exercise and Rehabilitation Sciences College of Life and Environmental Sciences University of Birmingham Birmingham U.K.; ^4^ Department of Innovative Technologies Dalle Molle Institute for Artificial Intelligence University of Applied Sciences and Arts of Southern Switzerland Manno Switzerland; ^5^ Centre for Performance Science Royal College of Music London U.K.; ^6^ Faculty of Medicine Imperial College London London U.K.

**Keywords:** pain location, pain extent, musicians, pain drawings

## Abstract

**Background and Aims:**

According to existing literature, musicians are at risk of experiencing a range of painful musculoskeletal conditions. Recently, a novel digital technology was developed to investigate pain location and pain extent. The aim of this study was to describe pain location and pain extent in musicians using a digital method for pain drawing (PD) analysis. Additionally, the association between PD variables and clinical features were explored in musicians with pain.

**Methods:**

One hundred and fifty‐eight musicians (90 women and 68 men; aged 22.4 ± 3.6 years) were recruited from Swiss and U.K. conservatories. Participants were asked to complete a survey including both background musical information and clinical features, the QuickDASH (QD) questionnaire, and the digital PDs.

**Results:**

Of the 158 participants, 126 musicians (79.7%) reported having pain, with higher prevalence in the areas of the neck and shoulders, the lower back, and the right arm. The mean percentage of pain extent was 3.1% ± 6.5%. The mean QD score was higher for musicians with pain than for those without pain. Additionally, the results indicated a positive correlation between the QD score and pain extent, and there were significant correlations between age and pain intensity, as well as between pain extent and pain intensity.

**Conclusions:**

The high prevalence of pain among musicians has been confirmed using a digital technique for PD acquisition and analysis. In addition, positive correlations between pain extent and upper limb disability have been demonstrated. Our findings highlight the need for effective prevention and treatment strategies for musicians.

## Introduction

The training needed to reach and maintain the highest levels of performance can expose musicians to a wide range of musculoskeletal health problems. Indeed, the acquisition and improvement of performance skills have been shown to expose musicians’ bodies, continuously and repeatedly, to contorted positions and unnatural movements.^1^ Not surprisingly, musicians are vulnerable to developing musculoskeletal disorders^2^, ^3^, ^4^ and to experiencing a range of physical problems, such as pain, weakness, and numbness that can affect how and how much they make music.^2^, ^3^, ^4^, ^5^

Although there are sporadic historical cases of scientific studies of the health of musicians,^6^, ^7^ the growth of performing arts medicine as a speciality field has occurred mainly over the past 30 years. In 1986, the concert pianist Gary Graffman published an article in the New York Times on his own focal dystonia and his difficulties in finding suitable treatment.^8^ Since then, large‐scale surveys of musicians have reported a high prevalence of performance‐related ill health.^1^, ^9^ This phenomenon was described by Zaza et al.^10^ as playing‐related musculoskeletal disorders (PRMDs) and includes any pain, weakness, numbness, tingling, or other physical symptoms that affect a musician's capacity to sing or to play their instruments at the level to which they are accustomed.

The existing research shows that PRMDs are commonly experienced both by professional musicians^9^, ^11^, ^12^ and by advanced music students.^13^, ^14^, ^15^ For instance, international surveys have reported the lifetime prevalence of PRMDs among orchestral musicians as between 39% and 87%,^10^, ^16^ with the majority of studies reporting figures in the upper portion of this range. Among advanced students, the prevalence is similarly between 32% and 89%.^17^

Pain, as a main complaint among musicians with PRMDs, has been investigated mainly in terms of its location,^18^, ^19^, ^20^ prevalence,^4^, ^21^, ^22^, ^23^ and sometimes intensity.^22^, ^24^, ^25^, ^26^, ^27^ The broad conception of pain found in the performing arts medicine literature is reflected in the variety of measures used to study it. For instance, investigations with musicians often rely on validated questionnaires for the general population, such as the Disabilities of the Arm, Shoulder, and Hand (DASH) Questionnaire, which measures upper‐extremity disability and symptoms,^4^, ^28^, ^29^ the Standardized Nordic Questionnaire, which measures pain location,^4^, ^21^, ^22^, ^23^, ^30^ or the SF‐12, which measure general physical and mental health.^28^, ^31^, ^32^ Bespoke surveys have also been constructed,^11^, ^13^, ^24^, ^33^ and interviews have been used to shed light on experiences of pain within the wider context of professional life.^34^, ^35^ In addition, some studies have employed physical tests specifically designed for musicians.^2^, ^11^, ^16^

Outside of the performing arts, recent advancements in technology have led to new digital methods of recording pain location and extent.^36^, ^37^ The method involves a user‐friendly interface made available on a tablet that contains a collection of body charts and customized software to analyze digital pain drawings (PDs). Using established protocols, people report their pain by drawing on different templates representing the human body (ie, body charts). Although not yet applied within the performing arts, digital PDs have become an important component in the assessment of pain and are now widely used to capture the location of pain and to assess its extent.^36^, ^38^, ^39^ Indeed, due to the lack of accuracy and reliability during the acquisition and analysis procedures of traditional paper body charts,^40^, ^41^, ^42^, ^43^, ^44^, ^45^ digital PDs are now recommended.^46^

This study sought to employ digital PDs for the first time in a large‐scale study of musicians’ pain. The purpose of this study was to investigate the location and the extent of pain in a sample of musicians using a digital tablet for PD acquisition. Additionally, the association between PD variables (i.e., pain location and pain extent) and musicians’ features were explored.

## Methods

This study forms part of a sample of musicians included in Musical Impact (32.7% of the entire sample), an interdisciplinary project investigating the health and well‐being of musicians studying and working in Europe.

Musical Impact has 3 core strands: (1) Fit to Perform explores the attitudes, perceptions, and behaviors of musicians toward health and well‐being, as well as their experience of chronic and acute health problems and their general fitness for performance; (2) Making Music investigates the physical and mental demands faced by musicians as they practice and perform; and (3) Better Practice examines strategies for promoting health effectively in music educational and professional contexts. This article focuses on Fit to Perform and, specifically, on self‐reports of pain extension and location using digital PDs.

### Participants

In total #bib158 musicians (90 women, 68 men) were recruited via e‐mail, institutional mailing lists, and social media from the Conservatory of Southern Switzerland (*n* = 68), Royal College of Music (*n* = 32), Royal Conservatoire of Scotland (*n* = 16), Royal Central School of Speech and Drama (*n* = 19), Royal Welsh College of Music and Drama (*n* = 13), and Southbank Sinfonia (SBS, *n* = 10). The mean age of the musicians was 22.4 years (SD ±3.6, range 17 to 41), 22.4 years (SD ±3.2) for women and 22.5 years (SD ±4.2) for men.

Inclusion criteria for participants were undergraduate and postgraduate professional music students (both women and men). Exclusion criteria included reports of clinically relevant conditions (i.e. any neurological or rheumatic disorders) or any cognitive disorders that may have influenced spatial perception and the completion of the PDs, none of which applied to the recruited participants.

At the time of the study, 59 participants were undergraduate students (mean age = 19.7, SD ±2.3; year 1, *n* = 42; year 2, *n* = 5; year 3, *n* = 6; year 4, *n* = 6), 89 were postgraduate students (mean age = 23.9, SD ±3.4; year 1, *n* = 62; year 2, *n* = 23; year 3, *n* = 4), and 10 were members of a professional ensemble on a 1‐year post‐graduation contract from the SBS (mean age = 25.4, SD ±2.1).

Participants were recruited between September 2014 and March 2015 and all participants received verbal and written information about the study. Informed written consent was obtained from all participants prior to data collection, and no payment was given in exchange for participation. The research was granted ethical approval by the Conservatoires UK Research Ethics Committee and was conducted according to ethical guidelines of the British Psychological Society.

### Materials

#### Background and Musical Information

General background questions elicited information on participants’ age, sex, nationality, principal instrument, career status, year of study, and institution. Information on height and weight, and the average number of hours per week devoted to practicing was also obtained.

#### QuickDASH

The QuickDASH (QD) is an 11‐item questionnaire used to measure physical function and symptoms in persons with musculoskeletal disorders of the upper limbs.^47^, ^48^ It is a reliable, shortened version of the 30‐item DASH Outcome Measure (Cronbach's α = 0.94). Respondents rate each item based on their experience over the preceding week on a 5‐point Likert‐type scale, increasing from 1 to 5 in level of difficulty/severity. Responses are averaged and then transformed into an overall disability/symptom score out of 100, where higher scores indicate greater disability. An optional module, specifically designed for athletes and performing artists, was also used in this study; it consists of 4 items, to which the same steps are applied to generate a separate score out of 100.

#### Digital Pain Drawings

Pain drawings were completed on a digital interface (Apple iPad 2) using a stylus pen designed for tablets (CS100B; Wacom, Vancouver, WA, U.S.A.) and a commercially available sketching software (SketchBook Pro). The reliability of this novel approach to assess pain has been confirmed in both chronic patients and in cases of acute painful stimuli.^36^, ^37^

**Figure 1:**
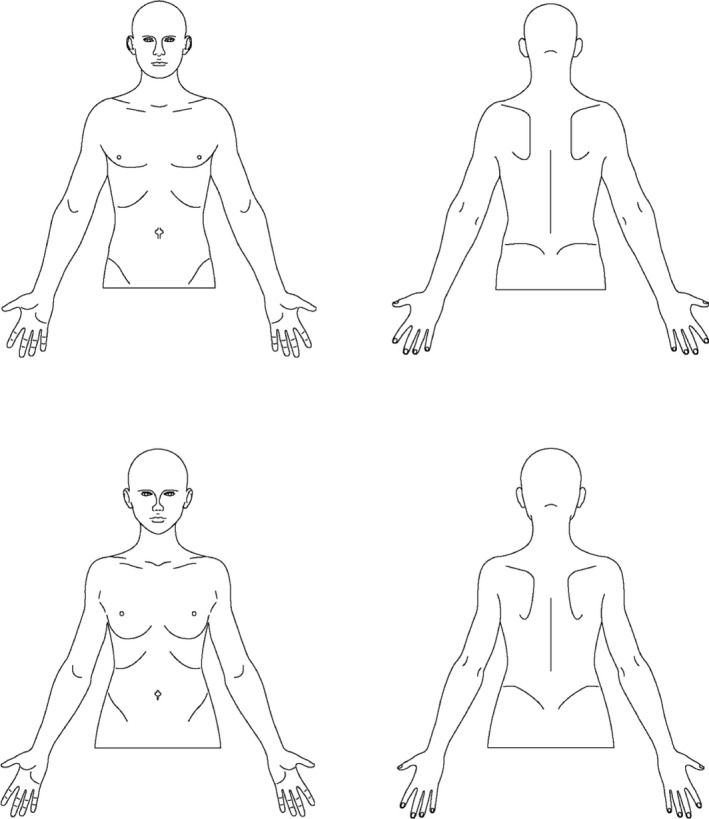
The template of male and female body charts (frontal and dorsal) in the sketching software.

A collection of male and female body charts of the upper body with 2 different views (frontal and dorsal) were used (Figure 1) and saved within the sketching software. All body charts have a closed perimeter and were reported on paintings with a size of 768 × 1,024 pixels. The type, size, and color of the pen strokes were standardized across all participants.

Using customized software for the analysis of PDs, pain extent, expressed as the number of pixels colored inside the frontal and dorsal body charts (the total area of pain for each participant), and pain frequency maps were computed. The pain frequency map is a function in which all the PDs are overlaid and analyzed simultaneously to indicate the most frequently reported location of pain across the entire sample. A color grid was used to illustrate the percentage of participants who reported pain in a specific area.^37^ This was computed for women and men separately.

### Procedure

Musicians were recruited in person and via e‐mail to take part in the study. Initially, participants were sent a detailed information sheet, and sessions were arranged to take place across each of the participating institutions, at a prearranged date and time. Following this, participants were asked to complete the survey with general background questions, as well as the QD. Following this assessment, after familiarization with the digital interface, participants were asked to complete the PD. Each participant was instructed verbally by an operator on how to complete PDs using a digital tablet. Two trained operators, each with one tablet, participated in the study and applied a protocol described in previous work.^37^ The following task was assigned: “Please shade on this body chart, using the stylus pen, where you felt your usual pain during the last week. Try to be precise and color every part of the body, independently from type and intensity of pain.” The session, including both the self‐report questionnaires and the PD acquisition, required approximately 20 minutes.

### Data Analysis

Distribution of the data was tested with the Shapiro‐Wilk test and non‐normally distributed data were observed; therefore, nonparametric tests have been employed for data analysis, as reported below. Descriptive statistics were used to investigate musicians’ features (ie, age, body mass index (BMI), practice hours, pain extent, pain intensity, QD score and QD score optional). The data were presented according to 3 different categories: symmetric playing position (SPP, *n* = 56), asymmetric playing position (APP, *n* = 78), and voice (*n* = 24). Instruments were allocated to SPP and APP categories according to the classification proposed by Wahlström‐Edling and Fjellman‐Wiklund^49^: SPP included bassoon, clarinet, oboe, percussion, piano/organ, and trumpet; APP included cello, double bass, flute, guitar, trombone, violin, and viola (see Discussion for further information on and justification of Wahlström‐Edling and Fjellman‐Wiklund's classification).

Using software developed and evaluated in previous work,^36^, ^37^ the following PD analyses were completed:

- Pain extent: Each pair of PDs (ie, frontal and dorsal) completed by the same musician was processed to quantify the total number of pixels colored inside the frontal and dorsal body charts. The pain extent was expressed as the percentage of the total body chart area.
- Pain frequency maps: All PDs were overlaid and analyzed simultaneously to indicate the most frequently reported location of pain across the entire sample. A color grid was used to illustrate the percentage of musicians who reported pain in a specific area. This was computed for the frontal and the dorsal body charts, and for women and men separately.
- Pain location: The body charts were divided into anatomical regions according to the Margolis rating,^45^ and the percentage of musicians reporting pain in specific body regions was presented using histograms.

The Wilcoxon rank‐sum test was used to verify whether the value of pain extent (expressed as a percentage) significantly changed according to sex. Spearman's correlation coefficients were computed to reveal possible associations between pain extent and musicians’ features (ie, age, BMI, practice hours, pain intensity, QD disability score, and score on the QD optional module for performing artists). The Wilcoxon rank‐sum test was used to test for differences in continuous variables (ie, age, BMI, practice hours, pain intensity, QD disability score, and score of the QD optional module for performing artists) in musicians with and without pain. Hypothesis tests with significance level α = 0.05 were used to identify significant correlations between observed variables. As several tests were performed, Bonferroni correction for multiple testing was applied.

Heat maps were generated to allow the visual comparison of pain frequency in different Margolis regions and for different groups of musicians. Frequency was computed as:

$$\frac{n_{1}+s/2}{n+s}$$

where *n* is the total number of musicians in a group, *n*_1_ is the number of those reporting pain, and *s* = 1 is a smoothing parameter correcting for small samples. The height of the rows in the heat maps is proportional to the size of each group of musicians.

All statistical analyses were carried out using the R language and environment for statistical computing (R Core Team 2015; R: A language and environment for statistical computing, R Foundation for Statistical Computing, Vienna, Austria; https://www.R-project.org).

## Results

### Descriptive Statistics

Table 1 shows descriptive features of the participants, including age, BMI, practice hours, pain intensity, QD disability score, and score on the QD optional module for performing artists, as well as pain extent. They are listed according to their playing posture and reported separately by sex. Following Wahlström‐Edling and Fjellman‐Wiklund,^49^ instruments classified as SPP (*n* = 56) included bassoon (*n* = 4), clarinet (*n* = 9), oboe (*n* = 6), percussion (*n* = 4), piano/organ (*n* = 24), and trumpet (*n* = 9). Those classified as APP (*n* = 78) included cello (*n* = 13), double bass (*n* = 5), flute (*n* = 12), guitar (*n* = 6), trombone (*n* = 5), violin (*n* = 25), and viola (*n* = 12). There were also 24 singers classified into a separate voice category.

**Table 1:** Descriptive Statistics

Variables	Median (IQR)			
	SPP	APP	Voice	Total
Age (years)	21 (5)	22 (6)	23 (4)	22 (5.3)
	f = 21 (6)	f = 22 (6)	f = 23 (4)	f = 22 (6)
	m = 21 (5)	m = 22 (6)	m = 25 (18)	m = 22 (5)
BMI	22 (5)	23.5 (7)	23.8 (4.3)	23.2 (6.1)
	f = 25.3 (7)	f = 24.1 (7)	f = 24.3 (5)	f = 24.4 (7)
	m = 22.3 (3)	m = 21.7 (5)	m = 23.4 (4)	m = 22 (4.3)
Practicing (hours)	29.5 (15)	32.3 (19)	11.7 (16.1)	30.6 (16.2)
	f = 28 (23)	f = 30 (24)	f = 11.5 (16)	f = 30 (23.6)
	m = 31 (12)	m = 34.5 (17)	m = 19.4 (18)	m = 32 (14)
Pain extent (%)	2.8 (7)	3.5 (6)	2.2 (3.2)	3.1 (6.5)
	f = 3.3 (12)	f = 3.7 (6)	f = 2.4 (3)	f = 3.6 (8)
	m = 2.3 (6)	m = 2.4 (6)	m = 1.2 (5)	m = 2.3 (6.3)
Pain intensity (1–5)	1 (1)	1 (1)	n/a	1 (1)
	f = 2 (2)	f = 1 (1)		f = 1 (1)
	m = 1 (1)	m = 1 (1)		m = 1 (1)
QD score (0–100)	5.7 (13)	2.3 (9)	n/a	2.3 (9.1)
	f = 9.1 (15)	f = 2.3 (11)		f = 4.6 (11.4)
	m = 2.3 (11)	m = 0 (6)		m = 1.1 (6.8)
QD score optional module (0–100)	0 (30)	0 (13)	n/a	0 (19)
	f = 0 (31)	f = 0 (16)		f = 0 (20.3)
	m = 0 (25)	m = 0 (13)		m = 0 (19)

Participants’ features (age, body mass index [BMI], practice hours) and clinical variables (pain intensity, QuickDASH [QD] disability score, score on the QD optional module for performing artists, and pain extent percentage). Values are expressed as medians and interquartile ranges (IQRs), reported according to their playing position (SPP, symmetric playing position [*n* = 56]; APP, asymmetric playing position [*n* = 78]; voice [*n* = 24]), and reported separately by sex (f, female; m, male).

Of the 158 musicians participating in the study #bib126 (79.7%) reported having pain in at least 1 Margolis anatomical region. Only 32 people (20.3%) reported having no pain.

**Figure 2:**
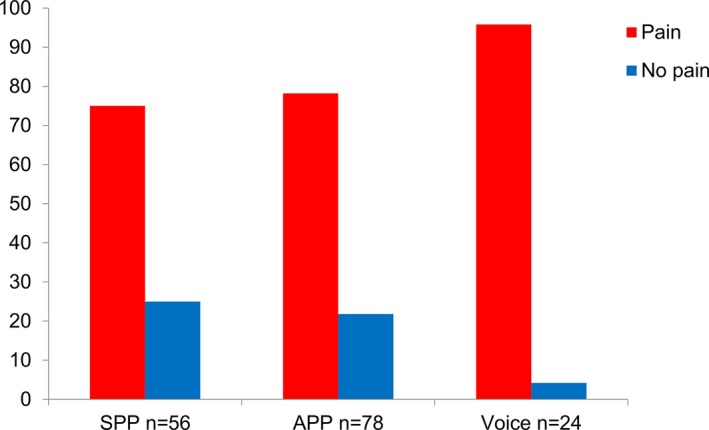
Prevalence of pain among musicians with Symmetric Playing Position (SPP, *n* = 56), Asymmetric Playing Position (APP, *n* = 78), and singers (Voice, *n* = 24).

Musicians with SPP and musicians with APP reported a similarly high number of complaints in at least 1 Margolis anatomical region, with a prevalence of 75% and 78.2%, respectively (Figure 2). On the other hand, singers reported the highest prevalence of complaints (95.8%), with 23 out of 24 reporting pain in at least 1 Margolis anatomical region. The mean of pain extent was 3.1% ± 6.5%.

### PD Analyses

**Figure 3:**
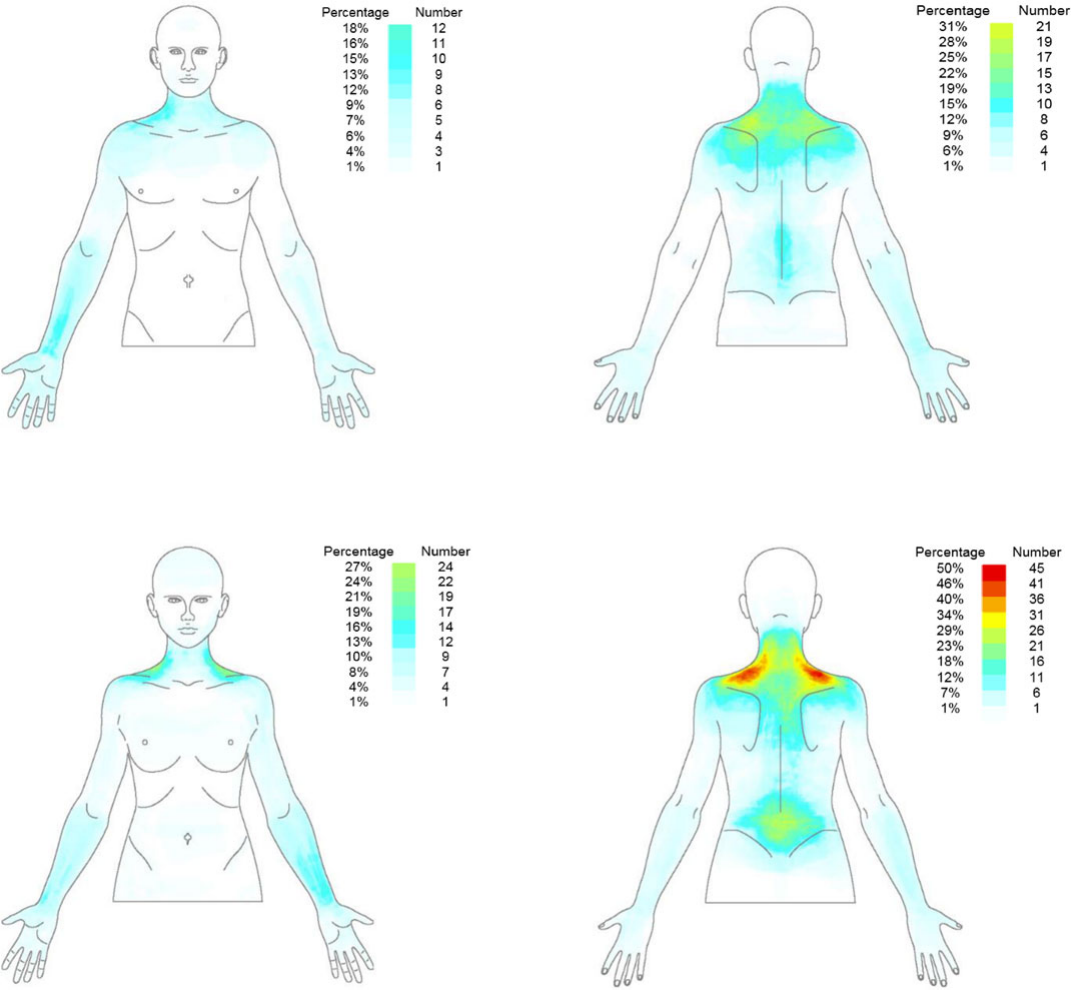
Pain frequency maps generated by superimposing the pain drawings of all participants included in the study (*n* = 158). Pain frequency maps have been generated for men and women separately and for both the dorsal and frontal view. The colour grid indicates both the number and the percentage of individuals that reported pain in the specific area. Dark red represents the most frequently reported area of pain.

**Figure 4:**
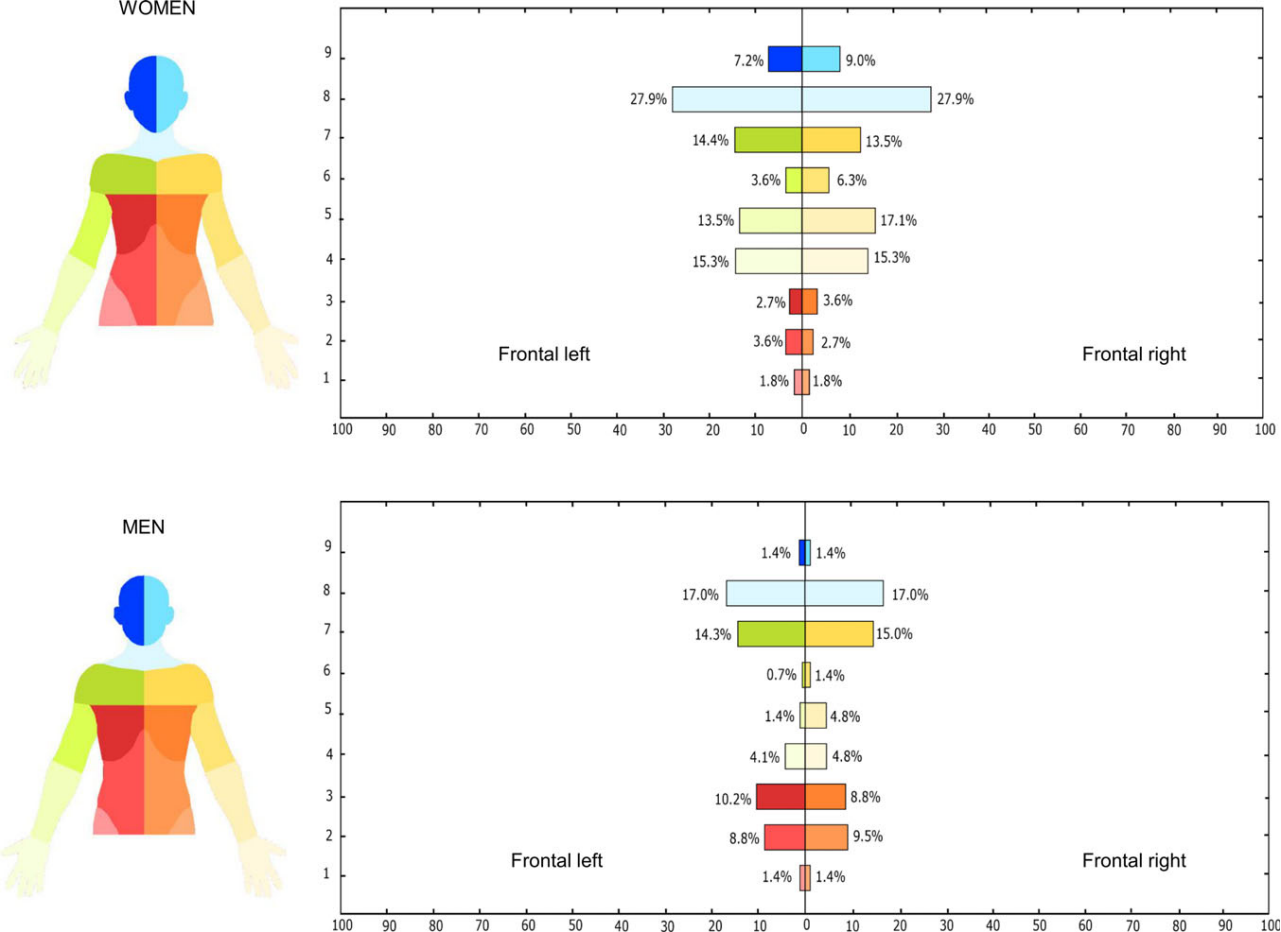
Pain location analysis which shows the percentage of individuals (*n* = 158) reporting pain in a specific body region of the frontal side. The regions of the body have been colour coded as displayed on the left side of the figure. The presence of the pain in a body region was confirmed when the pain drawing involved at least 10% of the body region area or where the number of pixels was greater than 60.

**Figure 5:**
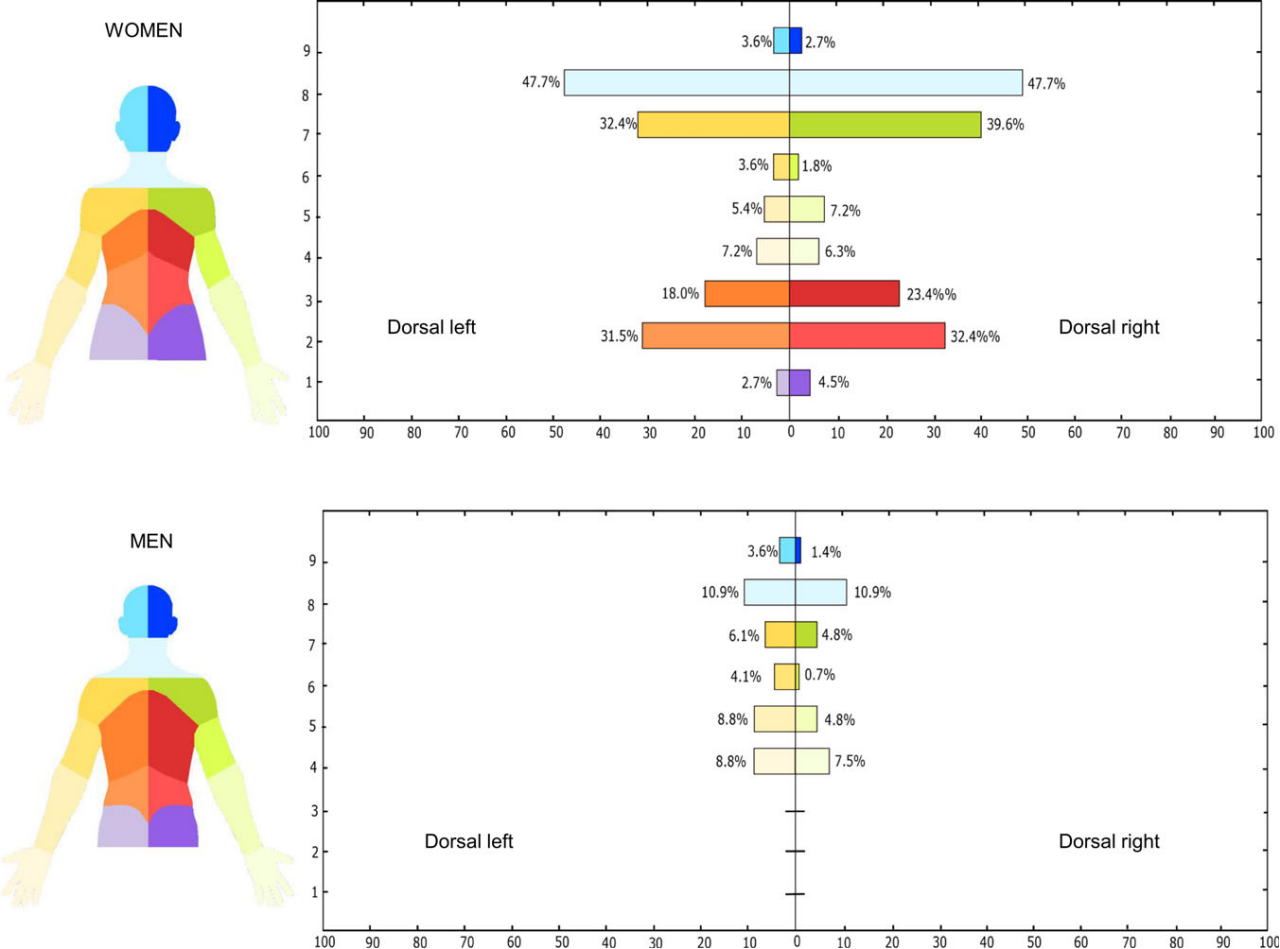
Pain location analysis which shows the percentage of individuals (*n* = 158) reporting pain in a specific body region of the dorsal side. The regions of the body have been colour coded as displayed on the left side of the figure. The presence of the pain in a body region was confirmed when the pain drawing involved at least 10% of the body region area or where the number of pixels was greater than 60.

Figure 3 illustrates the pain frequency maps for the full sample included in the study, whereas Figures 4 and 5 illustrate the pain location, where the perceived painful regions of the body for women and men for the frontal view (Figure 4) and dorsal view (Figure 5) of the body are reported.

The Wilcoxon rank‐sum test was run to determine if there were differences in pain extent between women and men. Distributions of pain extent for women and men were similar, as assessed by visual inspection. The results showed no statistical evidence of a relationship between pain extent and sex, and the pain extent was not significantly different between men and women.

### Correlational Analyses

The results of the correlational analyses between pain extent and musicians’ features (ie, age, BMI, practice hours, pain intensity, QD disability score, and score on the QD optional module for performing artists) are reported in Table 2.

**Table 2:** Correlation with Pain Extent

	*r*_s_	*P* value	*S*
Age	−0.038	0.319	682,090
BMI	0.068	0.198	612,590
Practice hours	−0.025	0.379	673,600
Pain			
Pain intensity	0.380	≤ 0.001*	407,840
QD			
QD disability score	0.459	≤ 0.001*	355,520
QD optional module score (module for performing artists)	0.424	≤ 0.001*	378,600

*Significant, *P* < 0.05.

*S*, Spearman's correlation coefficients between the pain extent computed from the pain drawings and musicians’ features; BMI, body mass index; QD, QuickDASH.

The Spearman correlation test to assess the relationship between the feature variables (ie, age, BMI, practice hours, pain intensity, QD disability score, and optional QD performing arts module score and pain extent showed no evidence of a relationship between age and pain extent, BMI and pain extent, nor practice hours and pain extent. Conversely, there was a significant positive correlation between pain extent and pain intensity (*P* ≤ 0.001). Furthermore, both the QD disability score and optional QD performing arts score increased with greater pain extent (*P* ≤ 0.001). The results of the relationships between all variables and pain presence in at least one Margolis region are reported in Table 3.

**Table 3:** Wilcoxon Rank‐Sum Test: Musicians with Pain vs. Musicians without Pain

	*P* value	*W*
Sex	0.061	3,499
Age	0.016	2,511.5
BMI	0.134	2,272.5
Practice hours	0.002*	2,700.5
Pain		
Pain intensity	n/a	n/a
QD		
QD disability score	< 0.001*	1,219
QD optional module score (module for performing artists)	< 0.001*	1,317.5

*Significant using Bonferroni’s correction for multiple comparisons (*P* value < 0.05/12 = 0.0042).

Results of the relationship between all variables and pain presence in at least one Margolis region. *W*, Wilcoxon rank‐sum test; BMI, body mass index; QD, QuickDASH.

The age of individuals reporting pain was significantly higher than the age of individuals not reporting pain (*P* = 0.016 < 0.01). However, the *P* value cannot be considered significant using Bonferroni's correction for multiple comparisons (*P* value < 0.05/12 = 0.0042), even though it is below the significance level of 0.05.

There was no statistical relationship between BMI and the presence of pain. However, the mean number of practice hours was significantly lower for people with pain (*P* = 0.002); similarly, the mean of both the QD disability score and the optional QD performing arts module score was higher for musicians reporting pain than for musicians without pain (*P* < 0.001).

### Heat Map

**Figure 6:**
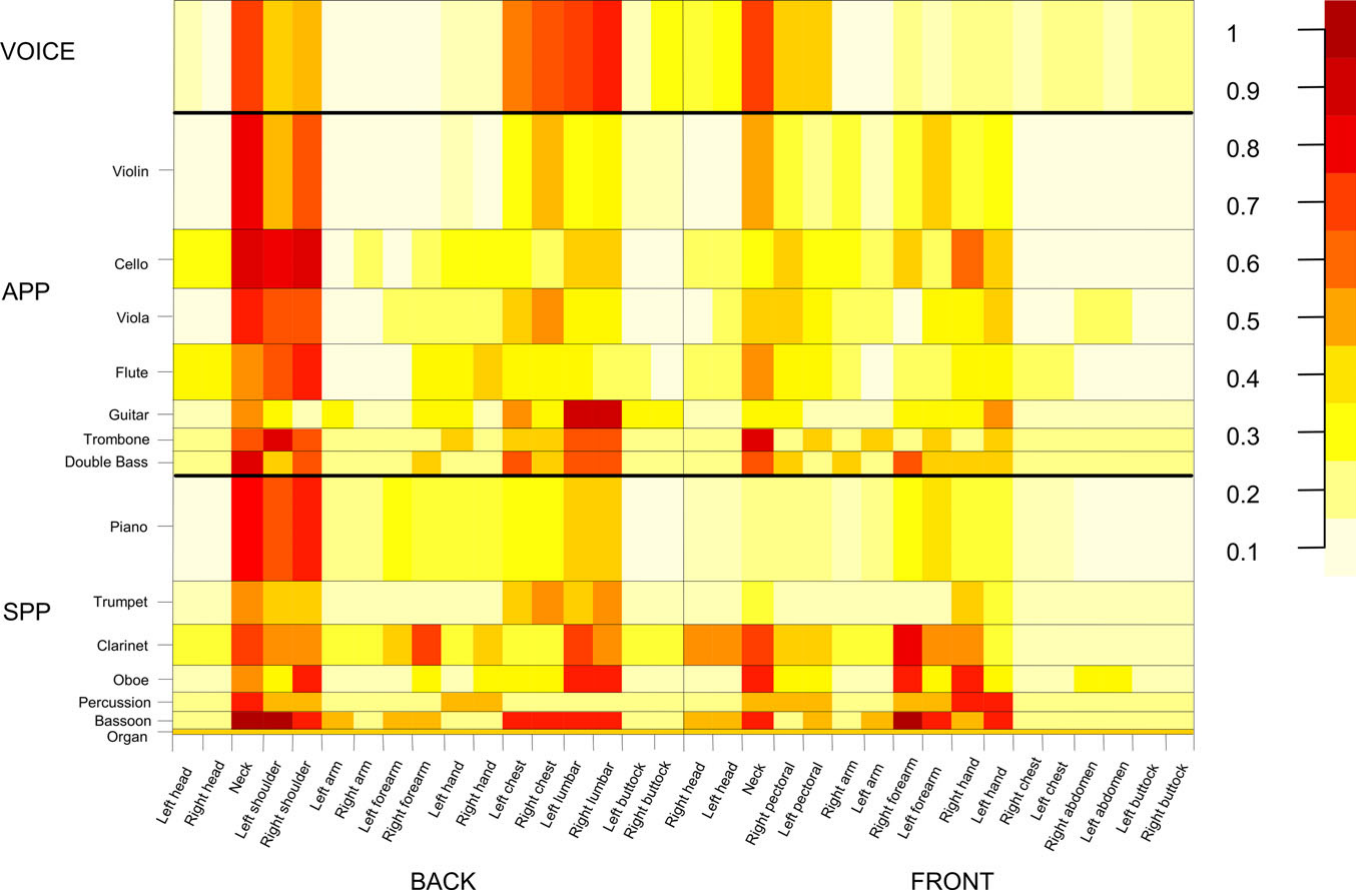
A heat map generated from pain location data of the three groups, which have been divided according to the playing posture (SPP = 56; APP = 78; Voice = 24). Dark red represents the most frequently reported pain location. The vertical dimension of the three categories depends on the samples size.

A heat map was generated to represent graphically the pain location among the 3 different groups: SPP, APP, and voice. The different colors correspond to the level of the measurement, with dark red representing the most frequently reported pain location. As seen in Figure 6, the heat map revealed that the neck and shoulder regions and, to a lesser extent, the area of the lower back were the most frequently affected areas.

## Discussion

This study examined performance‐related pain among musicians using analyses of a digital method for illustrating the location and the extent of pain. All participants were able to complete the PD. Although we did not formally assess the participants’ experience in completing their PD, informally participants revealed a high degree of ease in the ability to represent their pain. In addition, no one reported difficulties in identifying with the body charts, and many participants reported that the gender‐specific body charts were extremely important since they allowed a more accurate and individual expression of their’ pain.

In this study, we sought to include both location and extent of pain, which was straightforward to obtain from the digital PDs. Furthermore, direct data storage allows the PDs to be saved in an effective and accurate manner. Therefore, the assessment of the location and extent of pain was easy for our participants, offering a suitable and reliable instrument for use among healthcare practitioners and researchers.

Our findings are consistent with previous studies showing that the lifetime prevalence of musculoskeletal problems in musicians typically exceeds 50%, in most reports ranging between 62% and 93%.^4^, ^17^, ^31^

The observed pain extent in our sample was 3.1% of the total body chart area. Previous studies, which applied the same digital PD method, reported higher values of pain extent in patients with low back pain and whiplash.^37^, ^38^ This difference may be expected as both patient populations included those with chronic pain in which expanded areas of pain and widespread pain are common.

The individual PDs revealed large variability between musicians, yet collectively, as seen from the pain frequency maps presented in Figure 3, their reports of pain covered almost the entire upper part of the body (especially the dorsal part). Both the frontal and dorsal pain frequency maps clearly indicate that the neck and shoulder regions and, to a lesser extent, the lower back were the most frequently affected areas. In contrast, substantially fewer people reported pain in their pectoral and abdominal regions, although there was pain here for some musicians. A similar picture is provided by other studies that have investigated pain in musicians, where the regions with the highest prevalence of musculoskeletal symptoms were the shoulders, neck, and back.^4^, ^17^, ^29^

Recent studies showed that women are more inclined to experience pain than men.^4^, ^17^, ^29^, ^50^ Although there was no evidence of a relationship between sex and pain extent, the pain location analysis indicated that female musicians reported a higher occurrence of complaints than men, as illustrated in Figure 4 for the frontal aspect of the body and Figure 5 for the dorsal aspect. With regard to the frontal aspect, there is a prevalence of frequent pain in the area of the neck for both women and men, with an incidence of 27.9% and 17.0%, respectively. However, with respect to the other regions of the frontal aspect of the body, women and men presented with different locations of pain. While women reported a high prevalence of pain in the forearms and hands (eg, 17.1% in the right forearm) compared with men (eg, 4.8% in the right forearm), men reported more frequent pain in the chest and abdominals (ie, 10.2% in the left chest for men vs. 2.7% in the same region for women). Turning to the dorsal aspect of the body, the difference between women and men becomes more accentuated: female musicians reported a higher prevalence of complaints than men, especially in the neck (47.7%), right shoulder (39.6%), left shoulder (32.4%), and lower back (32.4% on the right and 31.5% on the left). Male musicians reported less pain, with a maximum of 10.9% of the men reporting pain in the neck.

Musicians are typically subject to monotonous performance positions that, depending on the instrument, often involves prolonged static use of the neck and shoulders, a repetitive use of joints in the upper extremity, or a combination of both. Although there was no statistical evidence of a relation between pain extent and practice hours, the mean number of practice hours was lower for people reporting pain in at least 1 Margolis area, suggesting that those with pain were less able to practice for long periods of time. At length, a daily practice routine accompanied by straining and repetitive movements can even degenerate into chronic health problems that may affect musicians irreparably. Many studies have shown that about 12% of musicians abandon their musical careers due to such problems.^1^, ^51^ Regarding age, our study revealed no evidence of a relation between age and pain extent. While comparison between the age of individuals reporting pain in at least 1 Margolis region and that of individuals not reporting pain (although not significant considering the number of tests performed) leaves room for the hypothesis that the former is higher than the latter as it produced a *P* value as low as 0.016. This could be attributed to the fact that a possible alteration of anthropometric characteristics could be developed after several years of practice. For example, the hand span or even the posture itself could be modified due to continuous stretching of ligaments, tendons, and muscles. Moreover, it has been demonstrated that the risk factors for the development of pain in musicians include (1) physical factors of the individual, such as age, sex, anatomical individualities (ie, joint laxity, arm and hand size), physical condition, and muscle conditioning and (2) music‐related factors, such as technique, posture while practicing, support of the instrument, duration of practice, change of instrument, playing time and intensity, and the repertoire itself.^9^, ^51^, ^52^

Considering the extreme physical demands of performance, musicians can be seen as athletes of the upper body. Investigations among musicians have revealed a difference between the instrumental groups in this respect and have demonstrated, for instance, that string players are more likely to experience pain than woodwind players.^4^, ^14^, ^23^, ^50^

Several instruments, such as the flute, guitar, violin, and viola, oblige the musician to adopt asymmetric playing positions.^49^, ^50^ With these instruments, players are required to elevate one or both arms, which in turn demand a constant static work of the muscles to steady the scapula and shoulder joint. Furthermore, they are required to rotate and turn the head, or keep an asymmetric posture with their lower back rotated to one side. In the meantime, repetitive movements with the arms and fingers are normally performed with a constant interaction between rapidity and precision.^49^ Conversely, other instruments, such as the clarinet, oboe, and piano, require more symmetric playing positions, with both arms nearby the body and the head straight. However, in order to play these instruments, a static and repetitive load on the arms and neck–shoulder muscles are still necessary.^4^, ^49^

In order to analyze differences in terms of pain prevalence among different instrumental groups, we used the classification of symmetry and asymmetry according to Wahlström‐Edling & Wiklund's study of musculoskeletal disorders and playing postures among music teachers.^49^ We employed an additional category for voice, due to the specific characteristics of their musical practice, where interestingly, our results showed the highest prevalence of pain among the 3 groups. This finding may be attributed to the fact that singers may experience an overuse of the vocal tract, and have to stand in static positions for long periods during both rehearsal and performance.

Nonetheless, when we take into account the distribution of pain in the various Margolis anatomic regions among the 3 groups (see Figure 6), the prevalence of pain in the neck, shoulders, and lower back was consistently high among all 3 groups. It is indeed remarkable that the majority of musicians seems a homogenous group in terms of pain location.

Regarding the pain extent, it should be noted that the highest value has been reported by musicians with an assymmetrical playing position (3.5%), which has been previously confirmed by other studies regarding the matter of asymmetry of musicians’ playing position.^4^, ^49^ Asymmetry of body position, which is a recognized issue in ergonomics for biomechanical risk assessments,^29^ involves playing with one or both arms elevated. Previous studies have shown that working with elevated arms could lead to muscle and tendon degeneration, which produces pain and distress.^49^, ^53^, ^54^, ^55^, ^56^

### Clinical Implications

In sum, singers and instrumentalists had a high and equally distributed frequency of pain, although singers reported a higher prevalence of symptoms than instrumentalists. These results could be employed to develop interventions of prevention initiatives for advanced musicians. These initiatives could consist of exercises tailored to specific body areas (namely, the neck, shoulders, and lower back) and generic exercises to enhance neuromuscular control to prevent pain, especially since low levels of physical conditioning and lack of exercise probably contribute to the appearance of musculoskeletal disorders in musicians.^2^ We can speculate that the lack of proper physical conditioning may play an important role in the high prevalence of pain observed in this study, and much needs to be done to prevent musicians from experiencing ongoing pain and disability.

### Methodological Considerations

To the best of our knowledge, our study is the first that used a digital platform to assess pain location and extent in musicians with reported upper quadrant complaints. The method proposed in the study represents an effort to optimize previous methods (ie, paper body charts) investigating pain among musicians.^11^ PDs can obtained directly from the patient, without any intervention from an investigator, which likely improves the quality and accuracy of the PD completion. The software used to evaluate the extent and location of pain removes estimation errors (ie, it is a deterministic system in which no randomness is involved), which possibly occur with visual‐subjective scoring methods.^37^, ^38^, ^45^ Moreover, the use of pixels allows the pain extent to be estimated accurately.

Finally, the method described in this study enables quantitative data to be extracted from the PDs, which can in turn be analyzed statistically.

However, although we had a relatively large sample size, it was not possible to find significant differences between the 3 groups (ie, SPP, APP, voice). It could be hypothesized that with a larger population in each group, other relationships could be found and more analyses could be conducted.

Additionally, psychological measures were not included in this study. However, it may be relevant in future studies to evaluate the association between pain reported in the digital PDs and psychological measures, in order to gain greater insight into the causes and personal significance of pain among musicians. A recent study on patients with whiplash‐associated disorders supported this approach and revealed that PD may be part of the psychological screening of patients with chronic painful conditions.^38^

Furthermore, future studies should examine whether the findings reported here are reproducible at a different playing level, including concert soloists and professional orchestral players.

## Limitations

There are limitations to be aware of when considering our findings. Firstly, PRMD is a collective term encompassing pain and several other distressing symptoms such as weakness, numbness, tingling, or other physical symptoms that affect the ability to play an instrument. In this study, we focused on pain only, as a main and specific complaint of PRMDs. A more comprehensive investigation considering other symptoms related to PRMDs may yield additional results furthering our understanding of the relevance of such symptoms in PRMDs.

Additionally, it is important to acknowledge that evidence indicates that the conscious sense of our body (i.e., the body image) and tactile acuity can be distorted in people with chronic painful conditions.^57^, ^58^ Although, the relationship between a distortion of the body image and the capacity to draw the pain experience on a body chart has never been investigated, it is reasonable to hypothesize that this condition may reduce the accuracy and the precision of the PD.

## Conclusions

The high prevalence of pain among musicians has been confirmed using digital PDs. In addition, a positive correlation between pain extent and upper limb disability has been demonstrated. Our findings highlight the need for effective prevention and treatment strategies for musicians.

## Author Contributions

C.C., M.B., and A.W. participated in the conception, design, and drafting of the manuscript. C.C., M.B., and L.S.A. performed and managed data collection. F.M. and L.A. analyzed the data. All authors discussed the results, contributing extensively to the work presented in this manuscript, and approved the final version.

## Conflict of Interest

The authors declare that the research was conducted in the absence of any commercial or financial relationships that could be construed as a potential conflict of interest.

## Research Ethics

The research was granted ethical approval by the Conservatoires UK Research Ethics Committee and was conducted according to ethical guidelines of the British Psychological Society. Informed consent was obtained from all participants, and no payment was given in exchange for participation.
